# Granulomatous Lymphocytic Interstitial Lung Disease Associated With Common Variable Immunodeficiency Presenting With Respiratory Failure

**DOI:** 10.7759/cureus.59037

**Published:** 2024-04-25

**Authors:** Yu Kusaka, Takehiko Oba

**Affiliations:** 1 Department of Respiratory Medicine, Ome Municipal General Hospital, Ome, JPN

**Keywords:** diffuse ground-glass opacities, acute respiratory failure with hypoxia, granulomatous lymphocytic interstitial lung disease, pulmonary hyalinizing granuloma, common variable immunodeficiency deficiency

## Abstract

This case study presents a rare occurrence of acute respiratory failure in a 17-year-old male diagnosed with common variable immunodeficiency (CVID) and granulomatous lymphocytic interstitial lung disease (GLILD), which typically have a gradual onset. The patient initially exhibited nonspecific symptoms such as dry cough and fever but quickly progressed to severe respiratory failure despite conventional treatments. Imaging showed extensive lung abnormalities, and blood tests revealed significantly low immunoglobulin levels, indicating an underlying immunodeficiency. Treatment with high-dose steroids and immunoglobulin replacement therapy resulted in a rapid and remarkable recovery of lung function. Lung biopsies confirmed the dual diagnoses of CVID and GLILD, emphasizing the challenge of diagnosing and managing GLILD in CVID patients. This case underscores the importance of early and aggressive intervention in improving outcomes for GLILD patients with acute respiratory distress.

## Introduction

Common variable immunodeficiency (CVID) is a primary immunodeficiency disorder characterized by a significant reduction in serum levels of immunoglobulin (Ig) G, IgA, and/or IgM, with a consequent increased susceptibility to infections. CVID presents a broad spectrum of clinical manifestations, not only limited to recurrent infections but also encompassing autoimmune phenomena, malignancies, and, notably, interstitial lung diseases (ILDs) [1,2]. Among ILDs, granulomatous lymphocytic ILD (GLILD) stands out as a distinct entity, predominantly associated with CVID, characterized by a combination of lymphoid proliferation and granuloma formation within the lung interstitium [3].

This case report underscores the critical role of high-dose steroid therapy in managing acute exacerbations of GLILD in a CVID patient, highlighting the potential for significant clinical improvement even in the face of rapidly progressing respiratory failure. Through this case, we aim to contribute to the growing literature on GLILD associated with CVID, offering insights into effective management strategies that can inform clinical practice.

## Case presentation

A 17-year-old male developed a dry cough and fever and visited a previous doctor. Despite being prescribed cough suppressants and clarithromycin, there was no improvement, and upon re-consultation, multiple pulmonary infiltrates were observed in both lung fields on chest X-ray and CT, leading to hospitalization with a diagnosis of pneumonia. After hospital admission, ampicillin/sulbactam and garenoxacin were administered for four days, but due to progressing respiratory failure, he was transferred to our hospital. He had no significant past medical history, no smoking history, and no allergies and was not on any regular medications. There was no significant medical history in his family. Upon admission, he was conscious, with vital signs showing a temperature of 37.0°C, pulse of 119 beats per minute and regular, blood pressure of 101/64 mmHg, and SpO2 of 86% (on 6 L/min oxygen via a reservoir mask). There was no conjunctival pallor or jaundice, no palpable cervical lymph nodes, and no heart murmurs, and fine crackles were heard in both lungs. No lower limb edema was observed.

Blood test results are shown in Table 1. There was no significant elevation in white blood cell count or C-reactive protein, indicating an absence of inflammatory markers in blood tests, but a marked decrease in immunoglobulins was noted. Arterial blood gas analysis under 6 L/min of oxygen showed hypoxemia with a PaO_2_ of 62.6 Torr. Lymphocyte subset analysis of peripheral blood showed normal ranges for CD3 and CD19 cell counts.

**Table 1:** Laboratory data on admission

Laboratory tests	Results	Normal range
White blood cell counts	3600	3300-9000 (/μL)
Red blood cell count	593	430-570 (×10^4^/μL)
Hemoglobin	16	13.5-17.5 (g/dL)
Platelet	20.4	14-34 (×10^4^/μL)
Neutrophils	73.6	55-70 (%)
Lymphocytes	18.3	20-40 (%)
Monocytes	7.8	2-8 (%)
Eosinophils	0	1-4 (%)
Basophils	0.3	0.5-1 (%)
Total protein	5.1	6.7-8.3 (g/dL)
Albumin	3.2	3.8-5.2 (g/dL)
Aspartate aminotransferase	58	10-40 (IU/L)
Alanine aminotransferase	33	5-45 (IU/L)
Lactate dehydrogenase	338	124-222 (U/L)
Blood urea nitrogen	29	8.0-20.0 (mg/dL)
Creatinine	0.77	0.47-0.79 (mg/dL)
Sodium	139	137-147 (mEq/L)
Potassium	4	3.5-5.0 (mEq/L)
Krebs von den Lungen-6	392	<500 (U/mL)
Surfactant proteins-D	83.2	<110 (ng/mL)
C-reactive protein	0.21	<0.3 (mg/dL)
Immunoglobulin G	385	861-1747 (mg/dL)
Immunoglobulin A	79	93-393 (mg/dL)
Immunoglobulin M	29	33-183 (mg/dL)
β-D-glucan	6.8	<20 (pg/mL)
Cytomegalovirus antigen	Negative	Negative
Cryptococcus antigen	Negative	Negative
Aspergillus antigen	Negative	Negative
T-SPOT	Negative	Negative
Antinuclear antibody	<40	<40
sIL2R	3650	122-496 (U/mL)
MPO-ANCA	Negative	Negative
PR3-ANCA	Negative	Negative
Lymphocyte subset		
T cell	76.9	％
B cell	3.7	％
CD4/8	2.6	
Arterial blood gas（O_2_ 6 L/min, non-rebreather mask）		
pH	7.41	7.35-7.45
PaCO_2_	36.6	35.0-45.0 (mmHg)
PaO_2_	62.6	80 (mmHg)
HCO_3_-	22.7	22-26 (mmol/L)
BE	-1	-3-2.3 (mmol/L)

**Figure 1:**
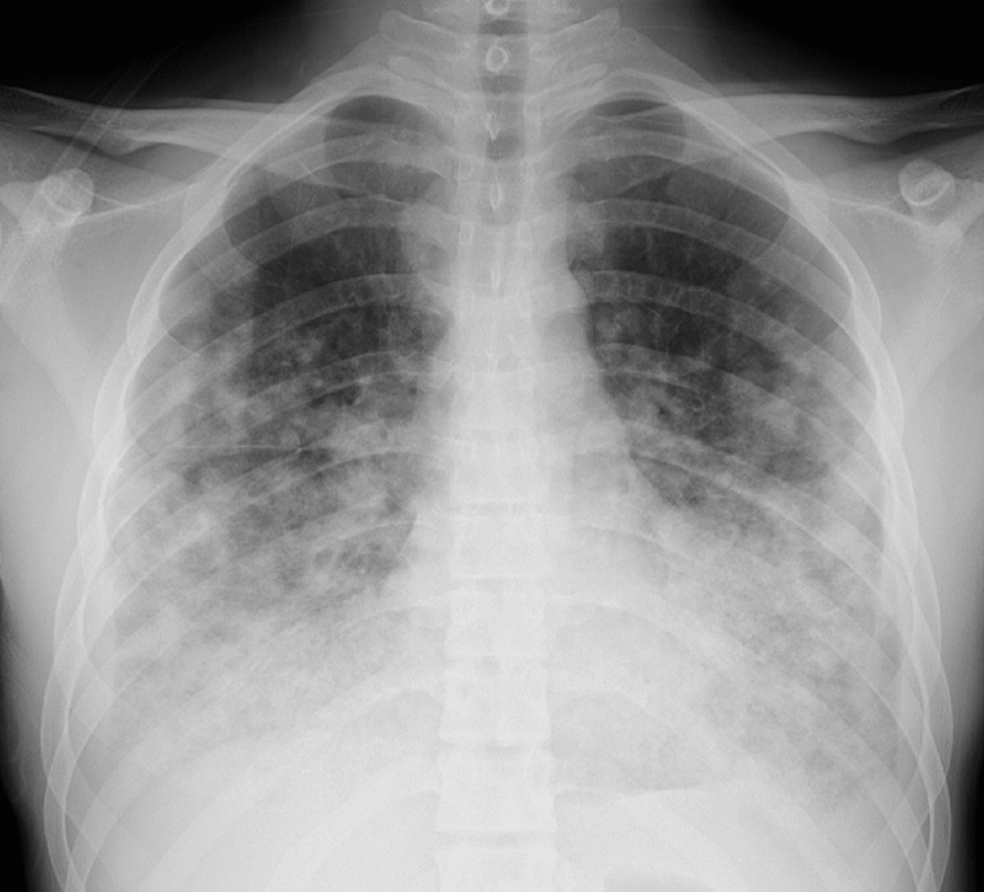
Chest X-ray Chest X-ray on admission showing ground-glass opacities and consolidation in both lower lung fields.

**Figure 2:**
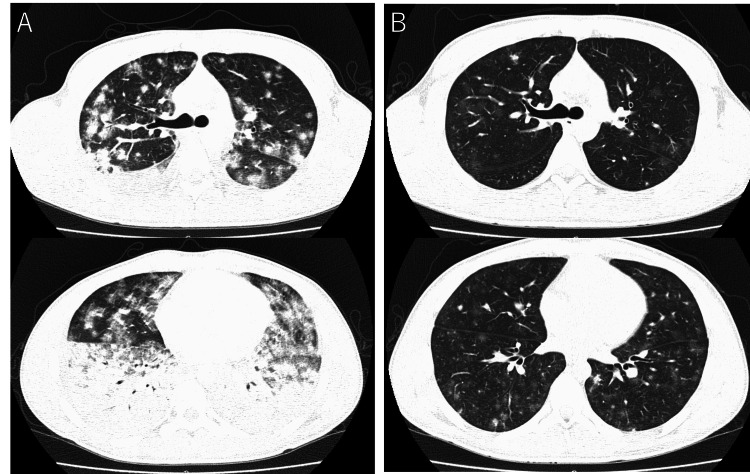
Chest CT A: On admission, chest CT showing progression of the diffuse ground-glass opacities and consolidation in both lower lung lobes. Some nodular shadows are also mixed. CT showed no hilar or mediastinal lymph node enlargement and a small amount of right pleural effusion. B: Hospital day 11, diffuse ground-glass opacities and consolidation in both lower lung lobes improved markedly.

Imaging findings are shown in Figures 1, 2A. Initial chest radiography (Figure 1) revealed patchy infiltrates and ground-glass opacities predominantly in the lower fields of both lungs. Chest CT (Figure 2A) showed diffuse infiltrates and ground-glass opacities dominantly on the dorsal side of both lower lobes. The shadows varied, with some nodular opacities intermixed and no hilar or mediastinal lymph node enlargement, and a small amount of right pleural effusion was observed.

**Figure 3:**
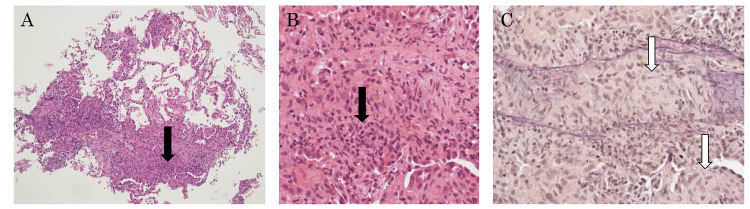
Histopathologic findings of a lung biopsy A: HE is staining x100, B: HE is staining x400, C: EVG staining x400 There was lymphocytic infiltration (A and B arrows) and granuloma (C arrows) in the pulmonary interstitium. There was no evidence of infection, including antimicrobial or fungal infections, or malignancy, such as lymphoma.

Given the significant decrease in immunoglobulins, a replenishment of immunoglobulins at 5 g/day for three days was performed. Considering the possibility of bacterial pneumonia and pulmonary cryptococcosis, administration of meropenem 3 g/day, levofloxacin 500 mg/day, and fluconazole 400 mg/day was initiated. As respiratory failure further progressed on the second day of illness, high-dose steroid therapy with methylprednisolone 1000 mg/day for three days was started. Blood and sputum cultures were obtained before initiating antibiotic treatment and were negative. On the fourth day, both respiratory status and imaging findings improved, prompting a bronchoscopy for diagnostic purposes. Bronchoalveolar lavage fluid from the right B5 showed an increased lymphocyte count of 49%. Cultures for bacteria and fungi, as well as acid-fast bacilli tests from the lavage fluid, were negative. Following the culture results, antibiotic and antifungal therapy was discontinued. Pathological diagnosis from transbronchial lung biopsies taken from right B8a, B4a, and B2b is shown in Figures 3A-3C, respectively. The specimen from B2b showed diverse inflammatory cell infiltration mainly composed of lymphocytes in the alveolar septa, with granulomatous infiltration of macrophages observed in the alveolar spaces and septa, diagnosed as granulomatous vasculitis. No necrosis was observed, and acid-fast and Grocott's staining were negative. No images suggestive of bacterial pneumonia were found. The lesion in B8a similarly showed diverse inflammatory cell infiltration predominantly in the septa. Mild infiltration of inflammatory cells in the alveolar walls was observed, along with widespread swelling of type II pneumocytes.

The infiltrating lymphocytes were small in size, with CD3-positive T cells predominating and a few CD20, CD79a-positive B cells. There was no atypia, and lymphoma was not actively considered. The atypical cell population was not indicated in surface marker analysis. Epstein-Barr virus expressing mRNA (EBER)-positive cells were not observed, and lymphomatoid granulomatosis was considered negative.

There is a significant decrease in immunoglobulins, ruling out secondary hypogammaglobulinemia due to conditions such as nephrotic syndrome, protein-losing enteropathy, malignancy, a history of medication or radiation therapy, and HIV infection, through blood tests and a comprehensive CT and medical history. Additionally, the low serum IgM, IgG, and IgA levels, normal T-cell count, B-cell count exceeding 1% of lymphocytes, and late onset led to the diagnosis of CVID. Consultation and registration of clinical information with the Primary Immunodeficiency Database in Japan (PIDJ: http://pidj.rcai.riken.jp/) were performed according to the diagnostic flowchart, confirming the diagnosis of CVID. The lung lesions observed in this case did not suggest infections, malignancy, vasculitis, organizing pneumonia, sarcoidosis, etc. and were diagnosed as GLILD complicating CVID.

After high-dose steroid therapy, tapering and maintenance therapy with prednisolone (PSL) were initiated, gradually reducing to PSL 10 mg/day by discharge. A CT scan on the 11th day of illness (Figure 2B) showed significant improvement in the infiltrates and ground-glass opacities in both lungs. Currently, the patient is maintained on PSL 5 mg/day and regular supplementation of 10 g immunoglobulins, with no recurrence and disappearance of pulmonary shadows observed subsequently.

## Discussion

This case involves a young male diagnosed with CVID triggered by respiratory failure, exhibiting lymphocyte infiltration and granulomas in the lung's broad interstitium. GLILD is defined as "an interstitial lung disease occurring in patients with CVID, characterized by lymphocyte infiltration and granulomas in the lungs, after excluding other diseases as much as possible" [4]. It generally affects individuals between their 20s and 50s, more commonly in females, with about 70% having a history of immunodeficiency. The etiology of GLILD remains unclear [5].

Recent studies have highlighted the significant impact of GLILD on the morbidity and mortality of patients with CVID, stressing the importance of early detection and aggressive treatment to improve outcomes [6]. However, the etiology and optimal management strategies for GLILD remain subjects of ongoing research. Approximately 20% of CVID patients are estimated to develop GLILD, which is associated with a worse prognosis compared to CVID patients without GLILD [5]. Despite its prevalence and impact, GLILD often presents diagnostic challenges due to its nonspecific clinical and radiographic features, which can overlap with other pulmonary conditions [7].

The pathogenesis of GLILD within the context of CVID is not fully understood, though it is believed to involve dysregulated immune responses, leading to chronic inflammation and granuloma formation [6]. The complexity of its pathophysiology underscores the necessity for a multidisciplinary approach to diagnosis and management, integrating clinical, radiological, and histopathological findings [8].

While no standard treatment for GLILD has been established, oral glucocorticoids have been selected as the initial treatment [4]. However, many patients respond poorly to treatment, and recent accumulated data suggest that a combination of rituximab and azathioprine might be the most effective initial treatment [5,9-12]. Data on the prognosis of CVID and GLILD are very limited. Bates et al. conducted a retrospective analysis of 69 CVID patients, reporting that patients with GLILD had a significantly lower survival rate than those without GLILD [13]. Untreated GLILD has been reported to lead to progressive respiratory dysfunction and respiratory failure [14]. The presence of fibrosis has also been reported as a poor prognostic factor [15].

To date, there have been no reports on cases of GLILD presenting with acute respiratory failure in its acute onset form. The notable response to steroid treatment in our case, despite the diffuse nature of the disease, suggests that diffuse ground-glass opacities during acute onset and increased lymphocytes in bronchoalveolar lavage fluid indicate a good steroid response, implying a well-maintained disease state over the long term, despite the unclear cause of the acute onset. With bimonthly supplementation of 10 g of immunoglobulins and daily administration of 5 mg PSL, the pulmonary lesions have remained stable for about six years, though careful monitoring is advised.

## Conclusions

This case study underscores the rarity of acute respiratory failure associated with CVID and GLILD, challenging traditional perceptions of their presentation. Swift intervention with high-dose steroids and immunoglobulin replacement therapy resulted in significant clinical improvement, emphasizing the critical role of early recognition and aggressive treatment in managing acute exacerbations of GLILD within the context of CVID.

Overall, these findings highlight the importance of heightened clinical awareness and prompt diagnostic consideration of GLILD in patients with acute respiratory distress and underlying immunodeficiency, contributing valuable insights into the management strategies and clinical outcomes of this rare and challenging condition.
